# Sepsis and septic shock – an observational study of the incidence, management, and mortality predictors in a medical intensive care unit

**DOI:** 10.3325/cmj.2020.61.429

**Published:** 2020-10

**Authors:** Vesna Vucelić, Iva Klobučar, Branka Đuras-Cuculić, Ana Gverić Grginić, Carmen Prohaska-Potočnik, Ines Jajić, Željko Vučičević, Vesna Degoricija

**Affiliations:** 1 Department of Medicine, Intensive Care Unit, “Sestre Milosrdnice” University Hospital Center, Zagreb, Croatia; 2 Department of Cardiology, “Sestre Milosrdnice” University Hospital Center, Zagreb, Croatia; 3 Department of Microbiology, “Sestre Milosrdnice” University Hospital Center, Zagreb, Croatia; 4 University of Zagreb School of Medicine, Zagreb, Croatia; The first two authors contributed equally.

## Abstract

**Aim:**

To prospectively determine the number of patients with sepsis and septic shock in a medical intensive care unit (ICU) using the Sepsis-3 definition; to analyze patients' characteristics, clinical signs, diagnostic test results, treatment and outcomes; and to define independent risk factors for ICU mortality.

**Methods:**

This prospective observational study enrolled all patients with the diagnosis of sepsis treated in the medical ICU of “Sestre Milosrdnice” University Hospital Center, Zagreb, between April 2017 and May 2018.

**Results:**

Out of 116 patients with sepsis, 54.3% were female. The median age was 73.5 years (IQR 63-82). The leading source of infection was the genitourinary tract (56.9%), followed by the lower respiratory tract (22.4%). A total of 35.3% of the patients experienced septic shock. Total ICU mortality for sepsis was 37.9%: 63.4% in patients with septic shock and 24.0% in patients without shock. Independent risk factors for ICU mortality were reduced mobility level (odds ratio [OR] 11.16, 95% confidence interval [CI] 2.45-50.91), failure to early recognize sepsis in the emergency department (OR 6.59, 95% CI 1.09-39.75), higher Sequential Organ Failure Assessment score at admission (OR 2.37, 95% CI 1.59-3.52), and inappropriate antimicrobial treatment (OR 9.99, 95% CI 2.57-38.87).

**Conclusion:**

While reduced mobility level and SOFA score are predetermined characteristics, early recognition of sepsis and the choice of appropriate antimicrobial treatment could be subject to change. Raising awareness of sepsis among emergency department physicians could improve its early recognition and increase the number of timely obtained specimens for microbial cultures.

According to the Sepsis-3 definition, sepsis is a life-threatening organ dysfunction caused by a dysregulated host response to infection (1). Almost 30% of patients treated in intensive care units (ICU) worldwide in 2012 had sepsis, with a mortality between 11.9% and 39.5%, varying across regions (2). It was the most expensive medical condition treated in the hospitals of the United States of America (USA) in 2011, costing 20.3 billion dollars or 5.2% of the total cost for hospitalizations (3).

Therefore, to improve patients' survival and plan resource allocation it is important to have up-to-date information about the incidence of sepsis for different regions. There have been no published data about the epidemiology of sepsis in Croatian hospitals since 2006 (4).

A revision of the sepsis definition in 2016 might have affected the disease incidence. To our knowledge, there are only a few studies reporting the incidences using the Sepsis-3 definition, and all of them retrospectively analyzed data sets collected for other purposes.

The aim of the present study was to prospectively determine the number of patients with sepsis and septic shock treated in the medical ICU in “Sestre Milosrdnice” University Hospital Center, Zagreb, using the Sepsis-3 definition; to analyze the demographic and social characteristics of the affected population, comorbidities, clinical signs, and laboratory test results at the time of admission, treatment methods and outcomes; as well as to compare the findings with our previous results (4).

## Patients and methods

### Study design

A prospective, observational, clinical, single-center study was conducted in the medical ICU of “Sestre Milosrdnice” University Hospital Center between April 1, 2017, and May 1, 2018 (13 months). The study enrolled all patients with the diagnosis of sepsis. The diagnosis was based upon the criteria from The Third International Consensus Definitions for Sepsis and Septic Shock (Sepsis-3) (1). The patients were treated according to the Surviving Sepsis Campaign: International Guidelines for Management of Sepsis and Septic Shock: 2016 (5).

### Patient management

According to standard clinical protocol, patient history data were recorded and physical examinations performed by two ICU physicians specialized in internal medicine and intensive care.

Venous blood samples were taken for standard laboratory testing. Blood for hematology tests was collected in tubes containing K3-EDTA and analyzed using the UniCel® DxH 800 Coulter® Cellular Analysis System (Beckman Coulter, Brea, CA, USA); for coagulation tests in tubes with sodium citrate and analyzed using the BCS HP System (Siemens Healthineers, Marburg, Germany); for standard biochemistry tests in plain tubes or in sodium fluoride/potassium oxalate-coated tubes for lactate assessment, and analyzed using ARCHITECT *c*8000 (Abbott Laboratories, Abbott Park, Chicago, IL, USA). Arterial blood samples for acid-base status assessment were collected in heparin-coated syringes and analyzed using ABL-90 FLEX Analyzer (Radiometer Medical ApS, Brønshøj, Denmark).

Before the administration of the first dose of antimicrobial drug, blood and urine samples for culture were taken. In the case of suspected lower respiratory tract infection, sputum or tracheal aspirate (for patients with endotracheal tube) were also obtained. All samples were collected and processed following UK Standards for Microbiology Investigations (6-8). The BACT/ALERT® 3D Blood Culture System (bioMérieux, Inc., Durham, NC, USA) was used for continuous blood culture monitoring and automated microbial detection. For isolated microorganisms, antimicrobial susceptibility testing was performed according to EUCAST guidelines (9).

After having achieved clinical recovery and laboratory test result improvement, with no need for further intensive care, the patients were transferred to one of the clinical wards within the Department of Medicine until hospital discharge.

### Data collection

Data were collected from patients’ history records, medical charts, and laboratory and microbiology test results by one of the researchers within a week of ICU admission and completed after the patients' hospital discharge or in-hospital death. Data recorded for each patient were demographics, social and epidemiological characteristics, chronic diseases, data about emergency department (ED) management, vital signs at ICU admission, laboratory test results at admission (complete blood count, prothrombin time, fibrinogen, acid-base status, lactate, urea, creatinine, electrolytes, glucose, bilirubin, alanine transaminase, aspartate transaminase, creatine kinase, lactate dehydrogenase, albumin, total proteins, C-reactive protein, procalcitonin), microbiology test results (blood, urine, sputum, tracheal aspirate cultures with antibiograms), type of antibiotic used, need for inotrope treatment, mechanical ventilation or hemodialysis; length of ICU stay, length of hospital stay and outcome. The Glasgow Coma Score (GCS) (10), Sequential Organ Failure Assessment (SOFA) (11), Acute Physiology and Chronic Health Evaluation II (12), Simplified Acute Physiology Score II (13), and Logistic Organ Dysfunction Score (LODS) (14) were calculated using data from the time of ICU admission. The SOFA score (11), the most widely used prognostic score for patients with sepsis, evaluates partial pressure of oxygen or fraction of inspired oxygen for ventilated patients, the GCS (10), platelets, bilirubin, creatinine, and mean arterial pressure (MAP), or administration of vasoactive agents. Patients who survived sepsis and were transferred to a clinical ward were referred to as ICU survivors, and those who died from sepsis in the ICU as ICU-non survivors. The additional data about 30-day mortality were obtained from medical records and by telephone contact with patients or their legal representatives. The anonymized data were manually imported into an electronic database in Microsoft Excel 2010 (Microsoft, Redmond, WA, USA) and checked one by one for possible errors. The study was approved by the Ethics Committee of the “Sestre Milosrdnice” University Hospital Center and performed in accordance with the principles of the Declaration of Helsinki (15). All patients or their legal representative signed informed consent for the participation before the enrollment.

### Statistical analysis

Categorical data are presented as absolute numbers (frequencies) and percentages. These were compared with the Pearson χ^2^ test. Continuous data are expressed as medians with interquartile ranges (IQR) or means with standard deviations (SD). The normality of distribution was tested with the Shapiro-Wilk test. The Mann-Whitney U test or *t* test were used to assess the significance of differences between the groups. All binary categorical and continuous variables were compared to the variable ICU survival using univariate binary logistic regression analysis. Significance was expressed as odds ratios (OR) and 95% confidence intervals (CI). Only variables significantly or marginally significantly associated to the variable ICU survival in univariate analyses were entered into the multivariate binary logistic regression (forward stepwise method), used to build a prediction model for ICU sepsis mortality. ICU survival analysis was performed with the log-rank test and depicted by Kaplan-Meier curves. Complementary subanalysis of 30-day sepsis mortality was also performed following the same algorithm. The level of statistical significance was set at *P* < 0.05. Statistical analysis was performed with IBM SPSS, version 25 (IBM Corporation, Armonk, NY, USA).

## Results

During a 13-month study period, 884 patients were treated in the medical ICU of “Sestre Milosrdnice” University Hospital Center, 116 (13.1%) of whom fulfilled the criteria for sepsis. Patients' demographic and social characteristics, as well as previously known chronic diseases are presented in Table 1. There was no significant difference in the number of men and women (*P* = 0.353) or their median age (*P* = 0.786). Chronic heart disease was more frequent among men (*P* = 0.036), while other chronic diseases were equally distributed between the sexes.

**Table 1:** The number of enrolled patients (N = 116) with sepsis; patients' sociodemographic characteristics and concomitant chronic diseases

Characteristic	
**Sex** (No, %)	
male	53 (45.7)
female	63 (54.3)
**Age** (median, IQR*)	73.5 (63.0-82.0)
male	73.0 (62.5-82.0)
female	74.0 (63.0-83.0)
**Comorbidities** (No, %)	
chronic cardiovascular disease	47 (40.5)
chronic renal insufficiency	45 (38.8)
diabetes mellitus	43 (37.1)
cerebrovascular disease	38 (32.8)
chronic pulmonary disease	22 (19.0)
malignant disease	22 (19.0)
**Permanently reduced mobility** (No, %)	58 (50.0)
**Previous accommodation** (No, %)	
home	70 (60.3)
nursery home	21 (18.1)
other clinical ward	22 (19.0)
other hospital	3 (2.6)

### Patient presentation to the emergency department

The majority of the patients presented to the ED during the winter months (49.1%). Median length of stay (LOS) in the ED was 4.0 hours (IQR 2.0-7.0), with no significant difference between ICU survivors and non-survivors. Nineteen (16.4%) patients were not initially recognized as having sepsis in the ED, which was associated with significantly more deaths in that group during ICU treatment (OR 6.59, 95% CI 1.09-39.75).

### Patient assessment in the intensive care unit

The mean MAP value at ICU admission was 87.1 ± 15.9 mm Hg among ICU survivors and 73.9 ± 19.2 mm Hg among non-survivors. Median heart rate among ICU survivors was 98 beats per minute (bpm) (IQR 80.0-119.5) and among non-survivors it was 117 bpm (IQR 95.0-126.75). Lower MAP and higher heart rate at admission were significant risk factors for death in the ICU in univariate logistic regression analysis (OR_MAP_ 0.96, 95% CI 0.93-0.98; OR_puls_ 1.02, 95% CI 1.001-1.03). Median body temperature for all patients was 36.5 °C (IQR 36.5-38.0), with no difference regarding survival. Calculated risk scores at the time of ICU admission are shown in Table 2.

**Table 2:** Median scores at the time of ICU admission for all patients, number of patients who developed septic shock and MODS at some point of ICU treatment, frequency of using different treatment methods in the ICU; comparison of all variables between ICU survivors and ICU non-survivors using univariate binary logistic regression*

	Total		ICU survivors		ICU non-survivors		Univariate binary logistic regression			
Variable							p	OR	95% CI upper	**95% CI lower**
**Scores at ICU admission** (median, IQR)										
GCS	13.0	11.0-14.0	14.0	13.0-15.0	11.0	10.0-13.0	<0.001	0.46	0.35	0.61
APACHE II	20.0	14.0-25.0	16.0	12.0-21.0	25.0	21.0-30.0	<0.001	1.32	1.19	1.47
SOFA	6.0	4.0-8.0	4.0	3.0-6.0	8.0	6.0-10.0	<0.001	2.23	1.69	2.94
SAPS	44.0	32.25-56.5	37.0	29.0-45.0	56.0	45.25-67.0	<0.001	1.10	1.06	1.14
LODS	7.0	4.0-12.0	5.0	3.0-8.0	12.0	8.0-14.0	<0.001	1.49	1.30	1.71
**Septic shock** (No, %)	41	35.3%	15	20.8%	26	59.1%	<0.001	5.49	2.40	12.56
**MODS** (No, %)	75	64.7%	34	47.2%	41	93.2%	<0.001	15.28	4.33	53.86
**Treatment** (No, %)										
empirical antimicrobial treatment only	51	44.0%	15	20.8%	36	81.8%	<0.001	17.10	6.59	44.40
antibiogram guided antimicrobial treatment	65	56.0%	57	79.2%	8	18.2%	<0.001	0.06	0.02	0.15
inotropes	41	35.3%	15	20.8%	26	59.1%	<0.001	5.49	2.40	12.56
mechanical ventilation	18	15.5%	5	6.9%	13	29.5%	0.002	5.62	1.84	17.15
hemodialysis	11	9.5%	7	9.7%	4	9.1%	0.910	0.93	0.26	3.37

*APACHE II – Acute Physiology and Chronic Health Evaluation; GCS – Glasgow Coma Score; ICU – intensive care unit; LODS – Logistic Organ Dysfunction Score; MODS – multiple organ dysfunction syndrome; SAPS – Simplified Acute Physiology Score; SOFA – Sequential Organ Failure Assessment; CI – confidence interval; IQR – interquartile range; OR – odds ratio.

### Diagnostic tests

**Figure 1:**
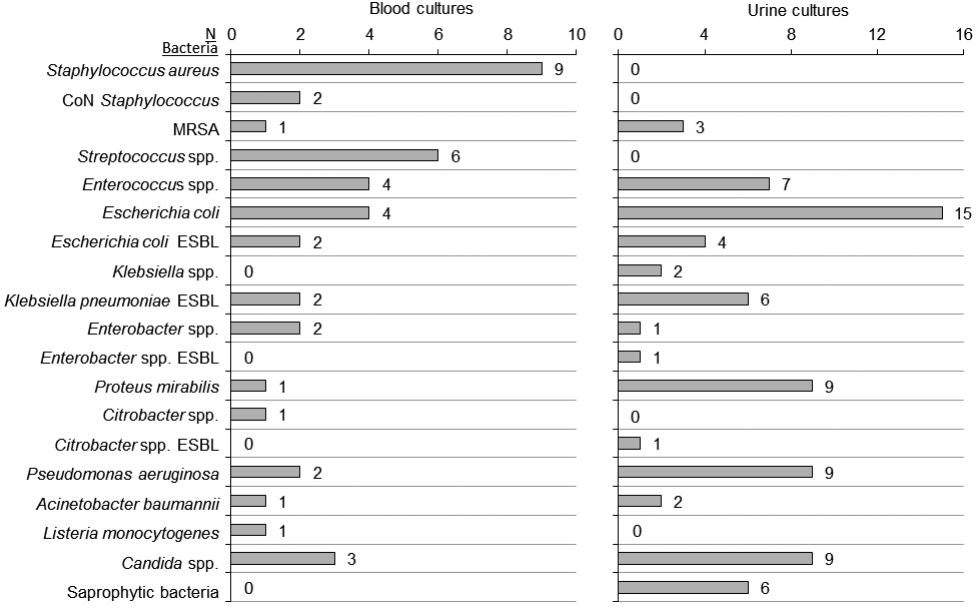
Bacteria isolated from blood and urine cultures. Numbers next to each bar represent the absolute number of patients having positive cultures with the growth of certain bacteria. CoN – coagulase-negative *Staphylococcus*, ESBL – extended-spectrum β-lactamase, MRSA – methicillin-resistant *Staphylococcus aureus*, spp – species (plural).

Blood samples for culture were taken from 95 patients (81.9%), 39 (41.1%) of whom were positive, with a total of 41 isolated microorganisms. Urine samples for culture were obtained from 97 patients (83.6%), with 62 (63.9%) positive results and 75 isolated microorganisms (11 cultures with multiple organisms). For 26 patients (22.4%) with suspected lower respiratory tract infection, sputum or tracheal aspirate were cultured. Twenty-two (84.6%) were positive, with 31 isolated microorganisms (7 cultures with multiple bacteria). Isolated bacteria from blood and urine specimens are presented in Figure 1.

**Figure 2:**
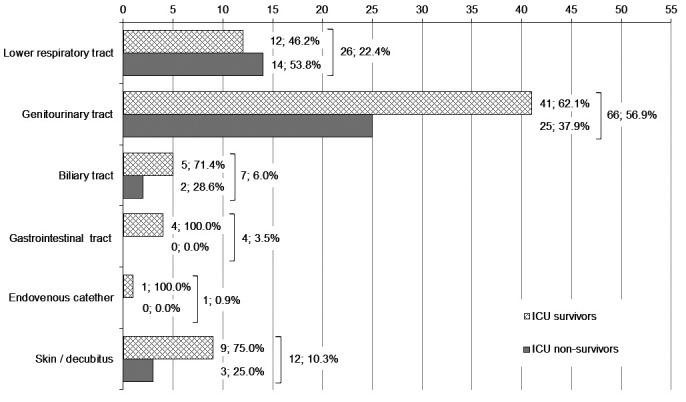
Primary sources of infection. Every source of infection is presented with two bars (pattern fill for intensive care unit [ICU] survivors, gray fill for ICU non-survivors). The absolute number and the percentage of ICU survivors/non-survivors within the total number of patients with a certain source of infection are presented alongside each bar. Square brackets are followed by the total number of patients with a certain source of infection and the percentage of these patients within the total number of patients in the study.

The sources of sepsis were located based on the laboratory and microbiology test results, as well as the results of body imaging studies (Figure 2).

Higher levels of platelets, prothrombin time, and pH at admission were associated with a lower risk of ICU death in univariate analysis (Table 3). The white blood cell count on days 1, 3, and 6 was significantly higher in women (p_d1_ = 0.002; p_d3_ = 0.002; p_d6_ = 0.035), but it was not recognized as a significant predictor of outcome among all patients.

**Table 3:** The list of the possible ICU mortality predicting variables as a result of univariate binary logistic regression; independent ICU mortality predictors derived from the multivariate binary logistic regression*

	ICU survivors		ICU non-survivors		Univariate binary logistic regression				Multivariate binary logistic regression			
Variable					p	OR	95% CI lower	95% CI upper	p	OR	95% CI lower	95% CI upper
Age (med; IQR)	69.5	59.25-82.75	77.5	69.25-81.75	0.070	1.03	0.998	1.06				
Sex (male) (No; %)	29	40.3%	24	54.5%	0.136	1.78	0.83	3.80				
CHD (No; %)	22	30.6%	25	56.8%	0.006	2.99	1.37	6.52				
COPD (No; %)	10	13.9%	12	27.3%	0.079	2.33	0.91	5.96				
CVD (No; %)	15	20.8%	23	52.3%	0.001	4.16	1.83	9.46				
CKD (No; %)	22	30.6%	23	52.3%	0.021	2.49	1.15	5.41				
DM (No; %)	23	31.9%	20	45.5%	0.146	1.78	0.82	3.85				
Reduced mobility (No; %)	24	33.3%	34	77.3%	<0.001	6.80	2.88	16.05	0.002	11.15	2.45	50.91
Unrecognized sepsis in ED (fever) (No; %)	8	11.1%	11	25.0%	0.055	2.67	0.98	7.27	0.040	6.59	1.09	39.75
GCS (med; IQR)†	14	13.0-15.0	11	10.0-13.0	<0.001	0.46	0.35	0.61	nt	nt	nt	nt
SOFA score (med; IQR)†	4	3.0-6.0	8	6.0-10.0	<0.001	2.23	1.69	2.94	<0.001	2.37	1.59	3.52
MAP (mmHg) (mean; SD)†	87.1	±15.9	73.9	±19.2	<0.001	0.96	0.93	0.98	nt	nt	nt	nt
Heart rate (bpm) (med; IQR)†	98	80-119.5	117	95.0-126.75	0.036	1.02	1.001	1.03				
T_ax_ (°C) (med; IQR)†	37.0	36.5-38.5	36.5	36.5-38.0	0.215	0.77	0.51	1.16				
Septic shock (No; %)	15	20.8%	26	59.1%	<0.001	5.49	2.40	12.56	nt	nt	nt	nt
MODS (No; %)	34	47.2%	41	93.2%	<0.001	15.28	4.33	53.86				
pH (med; IQR)†	7.38	7.32-7.46	7.37	7.26-7.43	0.034	0.08	0.01	0.83				
BE (med; IQR)†	-3.45	(-7.38)-0.28	-5.60	(-10.1)-(-1.3)	0.215	0.97	0.93	1.02				
RBC ( × 10^12^/L) (mean; SD)†	4.12	±0.85	4.46	±0.74	0.033	1.71	1.04	2.81				
Hemoglobin (g/L) (mean; SD)†	118.3	±23.6	126.4	±22.9	0.076	1.02	0.998	1.03				
Platelet count (x10^9^/L) (med; IQR)†	236.0	162.5-348.8	173.0	103.5-260.2	0.019	0.996	0.993	0.999	nt	nt	nt	nt
PT (%) (med; IQR)†	72.5	56.0-91.75	51.5	30.75-78.5	0.002	0.98	0.96	0.99				
Lactate (mmol/L) (med; IQR)†	1.5	0.9-2.3	2.1	1.4-4.8	0.152	1.77	0.81	3.88				
CRP (mg/L) (med; IQR)†	241.6	147.6-306.5	193.5	66.9-302.6	0.153	0.998	0.995	1.001				
Empirical antimicrobial treatment only (No; %)	15	20.8%	36	81.8%	<0.001	17.10	6.59	44.40	0.001	9.99	2.57	38.87
No of antibiogram guided antibiotics (med; IQR)	1	0.0-2.0	0	0.0-2.0	<0.001	0.10	0.04	0.25				
Mechanical ventilation (No; %)	5	6.9%	13	29.5%	0.002	5.62	1.84	17.15	nt	nt	nt	nt

*BE – base excess; bpm – beats per minute; CHD – chronic heart disease; CKD – chronic kidney disease; COPD – chronic obstructive pulmonary disease; CRP – C-reactive protein; CVD – cerebrovascular disease; DM – diabetes mellitus; ED – emergency department; GCS – Glasgow Coma Score; ICU – intensive care unit; MAP – mean arterial pressure; MODS – multiple organ dysfunction syndrome; PT – prothrombin time; RBC – red blood count; SOFA – Sequential Organ Failure Assessment; T_ax_ – body temperature measured axillary. IQR – interquartile range; med – median; nt – not tested in multivariate binary logistic regression, variables are part of SOFA score; OR – odds ratio, CI – confidence interval; SD – standard deviation.

†values measured at admission to ICU.

### Treatment

During the ICU treatment 41 patients (35.3%) developed septic shock and required vasopressor/inotrope therapy, 18 patients (15.5%) were mechanically ventilated, and 11 patients (9.5%) needed acute hemodialysis (Table 2).

Fifty-one patients received empirical antimicrobial treatment only (due to inability to culture and isolate specific microorganisms from obtained samples), with the mortality rate of 81.8%, and 65 patients were treated with antibiogram-guided antimicrobial treatment, with the mortality rate of 18.2% (Table 2).

### Outcome

*ICU outcome.* The median LOS in the ICU was 5.0 days (IQR 3.0-7.75) for all patients with sepsis. ICU survivors spent significantly more time in the ICU compared with non-survivors [6.0 (IQR 5.0-9.0) vs 3.0 (IQR 1.0-5.0) days; *P* < 0.001], as well as ICU survivors with septic shock compared with ICU survivors without septic shock [9.0 (IQR 6.0-12.0) vs 6.0 (IQR 4.5-8.5) days; *P* = 0.044].

**Figure 3:**
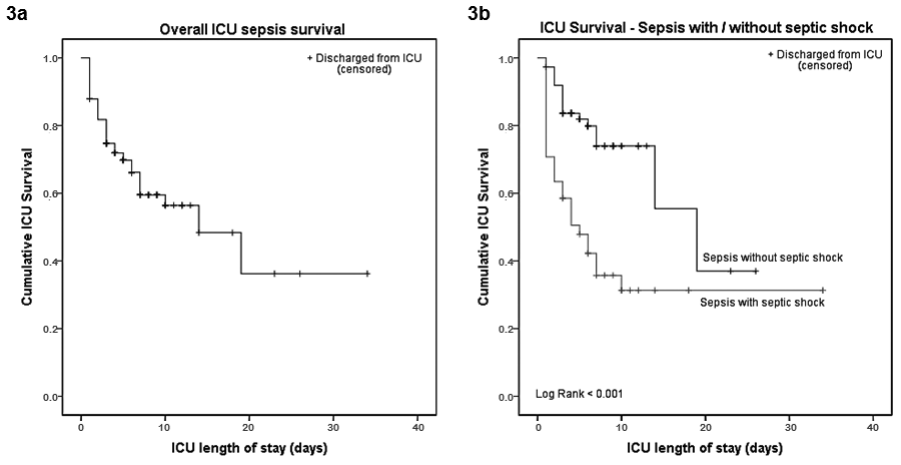
Kaplan-Meier curves for (**A**) overall intensive care unit (ICU) sepsis survival and (**B**) ICU sepsis survival depending on the presence of septic shock.

Forty-four patients died during ICU treatment (37.9%). ICU mortality rate for patients with sepsis and septic shock was 63.4%, while for patients with sepsis without septic shock it was 24.0%. Cumulative ICU survival for all patients with sepsis is depicted by the Kaplan-Meier curve (Figure 3A). There was a significant difference in survival depending on the presence of septic shock (Figure 3B). Almost half of ICU non-survivors with septic shock (46.2%) died during the first 24 hours of treatment.

All variables significantly associated with ICU survival in univariate analyses were entered into the multivariate logistic regression analysis using the forward stepwise method. The SOFA score, as part of the sepsis definition, was included despite being a composite variable. Variables contained in the SOFA score were not entered separately. All other calculated scores, despite their proven predictive power, were excluded from the analysis to ensure the independence of variables. Multivariate logistic regression analysis showed that the most notable risk factors for death among patients with sepsis in the ICU were reduced mobility level, failure to early recognize sepsis in the ED, higher SOFA score at admission, and inappropriate antimicrobial treatment (Table 3). Taken together, they form a predictive model for ICU mortality and significantly improve the predictive power of the SOFA score alone (AUROC_SOFA_ = 0.894 vs AUROC_model_ = 0.959) (Supplementary Figure(web extra material 1)).

**Figure 4:**
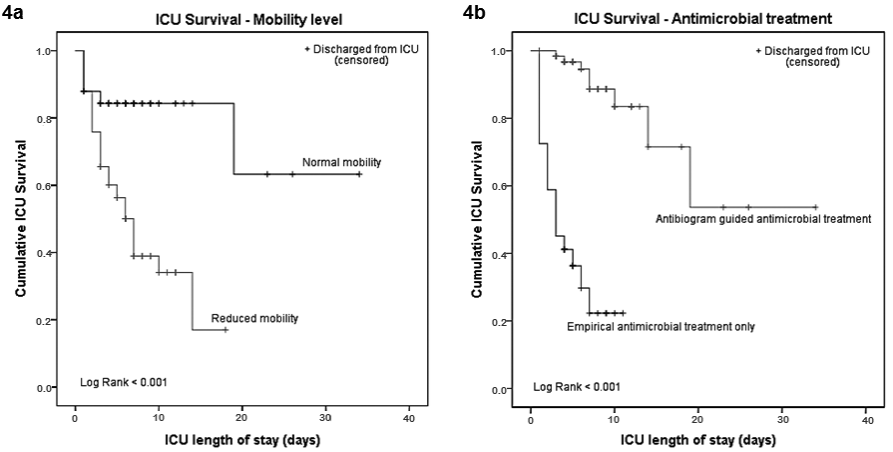
Kaplan-Meier curves for (**A**) intensive care unit (ICU) sepsis survival depending on mobility level, (**B**) ICU sepsis survival depending on appropriateness of antimicrobial treatment.

Kaplan-Meier curves for ICU sepsis survival depending on patient mobility and appropriateness of antimicrobial treatment, as the two most significant categorical predictors of death in the ICU, are presented by Figures 4A and 4B.

*Thirty-day outcome.* Fifty-six patients (48.3%) died during the first 30 days after the disease onset. Thirty-day mortality rate for patients with sepsis and septic shock was 75.6% and the rate for patients with sepsis without septic shock was 33.3%. The most important independent risk factors for 30-day mortality were reduced mobility level (OR 7.96, 95% CI 2.49-25.40), higher SOFA score at admission (OR 1.53, 95% CI 1.14-2.04), inappropriate antimicrobial treatment (OR 6.77, 95% CI 1.99-23.01), and prolonged prothrombin time at admission (OR 0.97, 95% CI 0.95-0.99).

## Discussion

Although the Sepsis-3 definition was published in 2016 (1), prospectively collected data about the epidemiology of sepsis using that definition are rarely published and this is the main value of the present study. Together with the results from previous research by Degoricija et al (4), this study also completes an overview of 18-year trends in the epidemiology of sepsis for “Sestre Milosrdnice” University Hospital Center.

Degoricija et al (4) published a complementary analysis of patients with sepsis treated in the ICU of “Sestre Milosrdnice” University Hospital Center between 2000 and 2005. Compared with the 314 episodes of sepsis during the 6-year period, the number of patients with sepsis substantially increased. The real increase may even be attenuated by the fact that the previous study was designed using the Sepsis-1 and -2 definitions, which are less specific than the Sepsis-3 definition (16) and, therefore, a wider range of patients was regarded as having sepsis.

Current patients with sepsis are older compared with those from our previous study [median age 73.5 (range 23-98) vs 71 (19-91) years], with no significant difference in sex distribution.

Urinary tract infections remain the leading source of infection (53.5% of all sources in 2000-2005, 56.9% in 2017), while lower respiratory tract infections (14.0% of all sources in 2000-2005, 22.4% in 2017) replaced skin and soft tissue infections as the second leading cause.

The percentage of the patients meeting the criteria for septic shock decreased from 43.9% in the previous study to 35.3% in 2017, probably due to the more rigorous Sepsis-3 definition of septic shock, which included the serum lactate level >2 mmol/L as a mandatory condition (1,16-19).

Although an increase in overall mortality of sepsis and septic shock was expected as a result of the definition changes (a smaller number of more seriously ill patients being defined as having sepsis or septic shock) (17-21), in the present study the percentage of ICU non-survivors decreased from 43.6% to 37.9% for sepsis in general, and from 75.4% to 63.4% for septic shock. This decrease may be explained by the improvement in treatment, but the mortality rates remain unacceptably high compared with high-income countries.

Literature review showed that only a few studies used the Sepsis-3 definition, all of them applying the definition retrospectively on patient data sets collected for other purposes (17,18,20-23). In the absence of prospective studies using the Sepsis-3 definition, we compared our results to those performed retrospectively.

An increase in the total number of patients with sepsis in the past years has also been reported in the United Kingdom (UK) (21) and Germany (24), although Vincent et al (19) consider this phenomenon controversial due to an increased awareness of sepsis among medical professionals and reporting bias.

The patients with sepsis in the present study were substantially older (median 73.5 years, IQR 63.0-82.0) compared with those from the UK (mean 63.3 ± 16.9 years) (21), USA (median 64.8 years, IQR 54-75) (18), Greece (mean 61.0 ± 17.1 years for sepsis ICU non-survivors) (22), and Turkey (median 69, IQR 55-79) (23). The proportion of men in the total number of patients with sepsis was between 44% and 65.5% (17,18,21,22). Shankar-Hari et al (21) reported the male sex as an independent risk factor for ICU mortality.

The other challenging finding is that urinary tract infections remain the leading source of sepsis during 18 years in Zagreb, Croatia, while all other studies from middle- and high-income countries report lower respiratory tract infections as the most frequent sepsis source, followed by abdominal/gastrointestinal infections and urinary tract infections (2,17,20-23,25,26). The present in-hospital results are consistent with the data from the former CroICU-net registry for 24 Croatian ICUs in 2006 (27), with urinary tract infections being the main source of sepsis in 30.4% of patients, followed by respiratory tract infections in 21.1% of patients. This phenomenon has not been clarified yet.

The percentage of patients meeting the criteria for septic shock in the total number of septic patients according to the Sepsis-3 criteria (35.3% in the present study) differs significantly between countries – from 19.9% in the UK (21) to 44.0% in Greece (22) and 48.4% in the Netherlands (17). The higher incidence of septic shock patients in the ICU reported in some studies may be explained by fewer ICU beds available, resulting in a concentration of more severely ill patients (19).

Data about the overall mortality of patients with sepsis as defined by the Sepsis-3 definition are scarce, mostly due to the retrospective character of the previous studies and the uncertainty about the total number of sepsis patients using patient cohorts selected for other purposes. Shankar-Hari et al (21) reported an overall sepsis ICU mortality of 22.4% in the UK and Papadimitriou-Olivgeris et al (22) 53.6% in Greece, compared with 37.9% in the present study. Septic shock ICU mortality in the USA was substantially lower compared with any European country (19.2%) (18). Lower septic shock mortality rates in comparison with this study (63.4%) were observed in high-income Western European countries [(Netherlands 38.9% (17), Germany 44.3% (20), UK 46.7% (21)], while higher mortality rates were reported in Turkey (75.9%) (23) and Greece (85.5%) (22). The difference between Croatian results and those from Western European countries may be explained by the significantly higher median age of our patients. There is a lack of adequate institutions for palliative care for older and bedridden persons in Croatia, who consequently do not receive appropriate care at their homes and frequently require hospitalization in institutions for acute treatment, being more often exposed to pathogenic microorganisms. An interesting finding from these retrospective studies is that the septic shock mortality was higher after the switch from the Sepsis-1 or -2 to the Sepsis-3 definition in the same set of patient data (17,20,23).

Independent risk factors for ICU mortality in this study were reduced mobility level, failure to early recognize sepsis in the ED, higher SOFA score at admission, and inappropriate antimicrobial treatment. Interestingly, these variables form a predictive model for ICU mortality that significantly improves the predictive power of the SOFA score alone. In contrast to the other predictors, information on the appropriateness of antimicrobial treatment is not available at the time of ICU admission, which reduces the importance of the model in clinical decision making. Still, the analysis of these predictors separately can be used to improve the patients' outcomes. While reduced mobility level and SOFA score are determinants of an individual patient, early recognition of sepsis in the ED and obtaining specimens for microbiologic cultures before the first administration of antimicrobial drug (to increase the number of positive cultures) depend on the physician's skills and the work organization in the ED, both of which can be changed to improve patients' outcomes.

Although failure to early recognize sepsis in the ED is a strong predictive factor for ICU mortality, in contrast to all other important ICU mortality risk factors from the present study, it does not have a significant impact on 30-day outcome.

It is noteworthy that the study presents the single-center experience, which may differ from the global trends in the epidemiology of sepsis. Compared with data from the registry-based studies in other countries, sample size is relatively small, which limited the data analysis. Although ICU sepsis survival was defined as the primary outcome in order to assess ICU performance, data about uniform long-term follow-up would be valuable for the analysis of sepsis epidemiology in general.

Despite the switch to the Sepsis-3 definition in 2016 (1), the number of patients with sepsis was increased compared with previous data (4). Sepsis patients were substantially older compared with those from other countries. Urinary tract infections remained the leading source of sepsis. A third of the patients experienced septic shock during the treatment, with an ICU mortality rate of 63.4%, while the ICU mortality rate for sepsis without shock was 24.0%. Independent risk factors for ICU mortality were reduced mobility level, failure to early recognize sepsis in the ED, higher SOFA score at admission, and inappropriate antimicrobial treatment.

Raising awareness of sepsis among physicians in the ED could improve its early recognition, increase the amount of timely obtained specimens for microbial cultures, and lead to administration of appropriate therapy in a timely manner.

*IQR – interquartile range.

## References

[1] Singer M, Deutschman CS, Warren Seymour C, Shankar-Hari M, Annane D, Bauer M (2016). The Third International Consensus Definitions for Sepsis and Septic Shock (Sepsis-3).. JAMA.

[2] Sakr Y, Jaschinski U, Wittebole X, Szakmany T, Lipman J, Ńamendys-Silva SA (2018). Sepsis in intensive care unit patients: worldwide data from the intensive care over nations audit.. Open Forum Infect Dis.

[3] Torio CM, Andrews RM. National inpatient hospital costs: the most expensive conditions by payer, 2011. Agency for Health Research and Quality, Rockville, Maryland. Healthcare Cost and Utilisation Project Statistical Brief #160, 2013. Available from: https://www.hcup-us.ahrq.gov/reports/statbriefs/statbriefs.jsp. Accessed: June 22, 2019.

[4] Degoricija V, Sharma M, Legac A, Gradišer M, Šefer S, Vučičević Ž (2006). Survival analysis of 314 episodes of sepsis in medical intensive care unit in university hospital: impact of intensive care unit performance and antimicrobial therapy.. Croat Med J.

[5] Rhodes A, Evans LE, Alhazzani W, Levy MM, Antonelli M, Ferrer R (2017). Surviving sepsis campaign: international guidelines for management of sepsis and septic shock: 2016.. Intensive Care Med.

[6] Public Health England. Investigation of blood cultures (for Organisms other than Mycobacterium species). UK Standards for Microbiology Investigations [B 37]. 2014. Available from: https://www.gov.uk/government/collections/standards-for-microbiology-investigations-smi#bacteriology. Accessed: June 22, 2019.

[7] Public Health England. Investigation of Urine. UK Standards for Microbiology Investigations [B 41i8.1]. 2016. Available from: https://www.gov.uk/government/collections/standards-for-microbiology-investigations-smi#bacteriology. Accessed: June 22, 2019.

[8] Public Health England. Investigation of Bronchoalveolar Lavage, Sputum and Associated Specimens. UK Standards for Microbiology Investigations [B 57i3.2]. 2016. Available from: https://www.gov.uk/government/collections/standards-for-microbiology-investigations-smi#bacteriology. Accessed: June 22, 2019.

[9] The European Committee on Antimicrobial Susceptibility Testing (EUCAST). Breakpoint tables for interpretation of MICs and zone diameters [version 6.0]. 2016. Available from: http://www.eucast.org/ast_of_bacteria/previous_versions_of_documents. Accessed: June 22, 2019.

[10] Teasdale G, Jennett B (1974). Assessment of coma and impaired consciousness. A practical scale.. Lancet.

[11] Vincent JL, Moreno R, Takala J, Willatts S, De Mendonça A, Bruining H (1996). The SOFA (Sepsis-related Organ Failure Assessment) score to describe organ dysfunction/failure. On behalf of the Working Group on Sepsis-Related Problems of the European Society of Intensive Care Medicine.. Intensive Care Med.

[12] Knaus WA, Draper EA, Wagner DP, Zimmermann JE (1985). APACHE II: A severity of disease classification system.. Crit Care Med.

[13] Le Gall JR, Lemeshow S, Saulnier F (1993). A new simplified acute physiology score (SAPS II) based on a European/North American Multicenter Study.. JAMA.

[14] Le Gall JR, Klar J, Lemeshow S, Saulnier F, Alberti C, Artigas A (1996). The logistic organ dysfunction system. a new way to assess organ dysfunction in the intensive care unit.. JAMA.

[15] World Medical Association (2013). World Medical Association Declaration of Helsinki: Ethical Principles for Medical Research Involving Human Subjects.. JAMA.

[16] Henning DJ, Puskarich MA, Self WH, Howell MD, Donnino MW, Yealy DM (2017). An emergency department validation of the SEP-3 sepsis and septic shock definitions with 1992 Consensus Definitions.. Ann Emerg Med.

[17] Driessen RGH, Van de Poll MCG, Mol MF, Van Mook WNKA, Schnabel RM (2018). The influence of a change in septic shock definitions on intensive care epidemiology and outcome: comparison of sepsis-2 and sepsis-3 definitions.. Infect Dis (Lond).

[18] Kashyap R, Singh TD, Rayes H, O’Horo JC, Wilson G, Bauer P (2019). Association of septic shock definitions and standardized mortality ratio in a contemporary cohort of critically ill patients.. J Crit Care.

[19] Vincent JL, Jones G, David S, Olariu E, Cadwell KK (2019). Frequency and mortality of septic shock in Europe and North America: a systematic review and meta-analysis.. Crit Care.

[20] SepNet Critical Care Trials Group (2016). Incidence of severe sepsis and septic shock in German intensive care units: the prospective, multicentre INSEP study.. Intensive Care Med.

[21] Shankar-Hari M, Harrison DA, Rubenfeld GD, Rowan K (2017). Epidemiology of sepsis and septic shock in critical care units: comparison between sepsis-2 and sepsis-3 populations using a national critical care database.. Br J Anaesth.

[22] Papadimitriou-Olivgeris M, Aretha D, Zotou A, Koutsileou K, Zbouki A, Lefkaditi A (2016). The role of obesity in sepsis outcome among critically ill patients: a retrospective cohort analysis.. BioMed Res Int.

[23] Baykara N, Akalin H, Arslantaş MK, Hanci V, Çağlayan Ç, Kahveci F (2018). Epidemiology of sepsis in intensive care units in Turkey: a multicenter, point-prevalence study.. Crit Care.

[24] Fleischmann C, Thomas-Rueddel DO, Hartmann M, Hartog C, Welte T, Heublein S (2016). Hospital incidence and mortality rates of sepsis: an analysis of hospital episode (DRG) statistics in Germany from 2007 to 2013.. Dtsch Arztebl Int.

[25] Sakr Y, Elia C, Mascia L, Barberi B, Cardellino S, Livigni S (2013). Epidemiology and outcome of sepsis syndromes in Italian ICUs: a multicentre, observational cohort study in the region of Piedmont.. Minerva Anestesiol.

[26] Jeganathan N, Yau S, Ahuja N, Out D, Stein B, Fogg L (2017). The characteristics and impact of source of infection on sepsis-related ICU outcomes.. J Crit Care.

[27] Gornik I, Gašparović V (2006). Severe sepsis and septic shock in Croatian ICUs. [Poster presentation] 26th International Symposium on Intensive Care and Emergency Medicine, 21-24 March 2006, Brussels, Belgium.. Crit Care.

