# Supplementary material for: Sepsis and septic shock – an observational study of the incidence, management, and mortality predictors in a medical intensive care unit
Source: Croat Med J. 2020 Oct;61(5):429–39. doi: 10.3325/cmj.2020.61.429 (PMC7684537; doi:10.3325/cmj.2020.61.429)
Supplement: Supplementary Figure 1 [file CroatMedJ_61_s001.pdf]

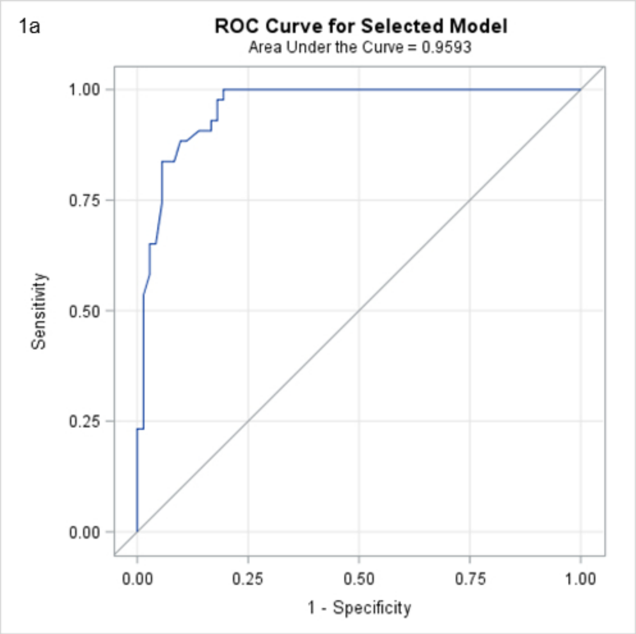
1a
ROC Curve for Selected Model
Area Under the Curve = 0.9593
Sensitivity
1.00
0.75
0.50
0.25
0.00
0.00
0.25
0.50
0.75
1.00
1 - Specificity

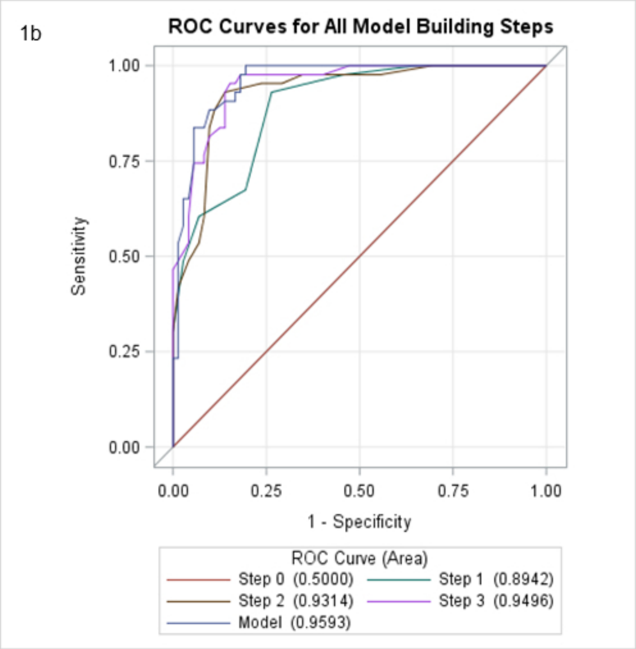
1b
ROC Curves for All Model Building Steps
Sensitivity
1.00
0.75
0.50
0.25
0.00
0.00
0.25
0.50
0.75
1.00
1 - Specificity
ROC Curve (Area)
Step 0 (0.5000)
Step 1 (0.8942)
Step 2 (0.9314)
Step 3 (0.9496)
Model (0.9593)
